# Comparison of the spatial-cognitive functions of dorsomedial striatum and anterior cingulate cortex in mice

**DOI:** 10.1371/journal.pone.0176295

**Published:** 2017-05-03

**Authors:** Tine Pooters, Annelies Laeremans, Ilse Gantois, Ben Vermaercke, Lutgarde Arckens, Rudi D’Hooge

**Affiliations:** 1 Department of Psychology, Laboratory of Biological Psychology, University of Leuven, Leuven, Belgium; 2 Department of Biology, Laboratory of Neuroplasticity and Neuroproteomics, University of Leuven, Leuven, Belgium; 3 Department of Biochemistry, McGill University, Montreal, Canada; Technion Israel Institute of Technology, ISRAEL

## Abstract

Neurons in anterior cingulate cortex (aCC) project to dorsomedial striatum (DMS) as part of a corticostriatal circuit with putative roles in learning and other cognitive functions. In the present study, the spatial-cognitive importance of aCC and DMS was assessed in the hidden-platform version of the Morris water maze (MWM). Brain lesion experiments that focused on areas of connectivity between these regions indicated their involvement in spatial cognition. MWM learning curves were markedly delayed in DMS-lesioned mice in the absence of other major functional impairments, whereas there was a more subtle, but still significant influence of aCC lesions. Lesioned mice displayed impaired abilities to use spatial search strategies, increased thigmotaxic swimming, and decreased searching in the proximity of the escape platform. Additionally, aCC and DMS activity was compared in mice between the early acquisition phase (2 and 3 days of training) and the over-trained high-proficiency phase (after 30 days of training). Neuroplasticity-related expression of the immediate early gene *Arc* implicated both regions during the goal-directed, early phases of spatial learning. These results suggest the functional involvement of aCC and DMS in processes of spatial cognition that model associative cortex-dependent, human episodic memory abilities.

## Introduction

The Morris water maze (MWM) is an established rodent model to investigate spatial learning and memory in laboratory animals [1]. The task requires rodents to associate a configuration of distal environmental cues with the position of a hidden escape platform [2–4]. During training, the animals gradually learn to swim away from the walls, and adopt more proficient search strategies [1,5,6]. They usually progress from non-spatial or repetitive strategies to cognitively demanding, but more efficient, spatial strategies [7,8]. Apart from the established hippocampal dependence of this task, striatal and prefrontal telencephalic regions have been implicated in MWM learning as well, although their involvement in the spatial-cognitive aspects of the task is still debated [9,10].

Both neural activation and lesion studies have shown that dorsal striatum, and more specifically its medial part, is crucial during the early phase of MWM training. Woolley and colleagues [10] reported increased activation of dorsomedial striatum (DMS) in mice during the first three days of training, and lesion studies showed that rats with DMS damage needed more time to acquire the hidden platform position [5,6,11,12]. Impaired performance in these animals might be, at least partly, attributed to disinclination or inability to acquire spatial search strategies. Increased tendency to keep on swimming along the wall of the pool (thigmotaxis) was indeed reported in rats with striatal lesions [5], rats with striatal dopamine depletion [13], PDE10A knockout mice with altered medium spiny neuron activity [14], and parkin-deficient mice [15].

There are indications that DMS interacts with other telencephalic structures to control spatial-cognitive functions. Notably, DMS is innervated by neurons in medial prefrontal cortex (mPFC), most prominently those in anterior cingulate cortex (aCC), as part of a distinct corticostriatal network [9,16–22]. Studies about aCC involvement in spatial learning and memory found that this brain region may play a role in storage and retrieval of remote memories (appr. 30 days after encoding) [23,24], but not in the acquisition of spatial information as aCC lesions failed to impair place learning [24,25,26]. However, reports failed to compare the involvement of mPFC and DMS in MWM learning [27–30]. A more recent study of Woolley and colleagues [10] reported that mPFC was activated in the mouse brain during early MWM place learning (the first 3 days of training), but not during late place learning. This early mPFC activation coincided with DMS activation, which definitely supports the involvement of the corticostriatal circuit in rodent and human spatial learning [10,31].

In the present study, we used complementary techniques to examine further the spatial-cognitive importance of aCC and DMS. In experiment 1, we compared the different effects of aCC and DMS lesions on specific, previously unexamined spatial-cognitive parameters, since lesion techniques remain the most established way to study regional brain function in mice [24,32,33]. In view of previous studies, DMS damage might impair MWM performance more profoundly than aCC lesions. Detailed analysis of the swimming paths allowed us to compare the involvement of each brain region in the use of cognitively demanding spatial search strategies. However, lesion studies are often confounded by functional compensation by other brain areas. In experiment 2, we therefore determined the involvement of these brain areas during early and late learning by visualizing regional expression of a known plasticity marker. Immediate early genes (IEG), in general, and particularly *Arc* (activity-regulated cytoskeleton-associated protein [34]; also known as Arg 3.1 [35]), are considered valid imaging tools to examine the neural substrate of learning and memory [36,37]. The localization of *Arc* mRNA and protein in activated dendrites [38–40], and their requirement for long-term potentiation maintenance and memory consolidation [41,42], implements this IEG in synaptic plasticity and memory [41,43–46]. To define the time dependence of this involvement, we compared regional activation between mice following short (2 days and 3 days, acquisition phase) and extended (30 days, overtrained phase) training. Quantified regional expression of *Arc* will be a read-out for neuroplasticity-related brain activation. More specifically, we used learning-dependent changes in *Arc* expression to visualize aCC and DMS involvement during MWM learning.

## Materials and methods

### Animals

Female C57BL/6J mice, 8 weeks of age, were purchased from Janvier Labs (Le-Genest-Saint-Isle, France) and group housed (6–9 mice per cage; cage dimensions: 46 cm x 27 cm floor, 23 cm high). Females were preferred for these experiments, despite possible influences of fluctuations in oestrus hormones [47–49], because in our experience, territorial fights have a more profound impact on behavioural read-outs in group-housed males. Food and water were available ad libitum, and mice were handled for 1 week (tail colouring) before the start of behavioural testing. Animals were housed in a temperature-controlled environment (22°C ± 2°C) that was maintained at 30–40% humidity, and kept on a 12-h light–dark cycle (lights on at 8:00 AM). Mice were tested behaviourally from 12 weeks of age and experiments were performed during the light phase. Study design and procedures approved according to European guidelines by Animal Ethics Committee of KU Leuven: https://admin.kuleuven.be/raden/en/animals-ethics-committee.

### Morris water maze protocol

Spatial learning was tested in the Morris water maze (MWM) with hidden platform. The maze consisted of a circular pool (150 cm diameter, 33 cm high), which was filled with opaque water (25 ± 1°C) to a depth of 16 cm, and an escape platform (15 cm diameter, 15 cm high) hidden 1 cm below the water surface in the middle of a fixed quadrant. The pool was situated in a room enriched with distal visual cues. Each trial began at one of four randomized starting locations by placing the mouse at the edge of the pool facing its centre. During trials, the experimenter remained seated at a fixed location. When a trial was not completed within 2 min, the mouse was guided to the platform and remained there for 15s. A training session consisted of four swimming trials during which the animal needed to find the position of the hidden platform. Trials in each session were separated by a 15-min break, and when two sessions were performed on a single day they were separated by 2h. Five consecutive training days were followed by 2 resting days. MWM navigation was recorded using Ethovision video tracking equipment and software (Noldus Information Technology, Wageningen, The Netherlands).

### Experiment 1: Effects of aCC and DMS lesions on MWM learning

#### Brain lesion procedure

Animals used in the lesion experiments (n = 34) were randomly assigned to one of the experimental groups: aCC lesions (n = 11), DMS lesions (n = 11), and a sham control group (aCC sham, n = 6; DMS sham, n = 6). Mice were anaesthetized by intraperitoneal injection of 5% chloral hydrate (1% of body weight) and positioned on a stereotaxic frame (Narishige scientific instruments, Tokyo, Japan). Corneal drying was prevented by ophthalmic ointment and 0.05 ml lidocaine (20 mg/ml) was injected subcutaneously before making an incision in the skin above the skull. Next, holes were drilled into the skull and bilateral electrolytic lesions were produced by applying direct current (0,5 mA for 30 s) through a tungsten-coated 0.2 mm needle (Teflon insulated Tungsten wire, advent research materials, Oxford, UK). Sham animals were submitted to the same surgical procedure but no current was delivered after placement of the electrode. Coordinates relative to Bregma were: (1) aCC, +1.0 mm anteroposterior (AP), ±0.2 mm mediolateral (ML), -1.5 mm dorsoventral (DV); (2) DMS, +0.2 and +1.2 mm AP, ±1.5 mm ML, -2.5 and -3.0 mm DV [50]. Post-surgery, each animal was treated with 5% glucose saline (10% of body weight) and placed on a heat pad in the recovery cage. Paracetamol (30 mg/ml) was administered in drinking water 24-h pre- and post-surgery. Animals were given 2 weeks of post-surgery recovery before starting the behavioural experiments.

Following behavioural testing animals were sacrificed by cervical dislocation. Brains were removed, snap-frozen in isopentane (Sigma-Aldrich, Germany) and stored at -80°C for further processing. Coronal sections (25 μm thickness) were cut using a cryostat (Microm HM 500 OM, Waldorf, Germany) and mounted on poly-L-lysine coated glass slides. Brain sections were Nissl stained with 5% thionin acetate (Alfa Aesar, Karlsruhe, Germany) followed by microscopic determination of lesion size and location (Optech Biostar microscopes, Germany).

#### Additional behavioural assessment

All animals were tested for motor defects and working memory impairments before starting MWM training. Motor coordination and balance were measured on an accelerating rotarod (MED Associates Inc., St. Albans, Vermont, US). Mice were first trained at a constant speed (4 rotations per minute, rpm; 2 min) followed by four test trials (3 min inter-trial interval, ITI). During test trials, rotation speed was increased from 4 to 40 rpm over 5 min (10 min ITI), and time on the rod before falling was recorded [51].

The Y-maze spontaneous alternation (SA) task assessed working memory functions. The animal was placed at the centre of three enclosed arms (30 cm long x 6 cm wide x 31 cm high) and was allowed to freely explore the arms during 10 min. Alternation behaviour was confirmed as soon as the mouse entered three different arms consecutively. The percentage of spontaneous alternations (%SA) was calculated by the formula [(sum alterations / sum arm entries– 2) x 100] [52,53], and the number of total arm entries was considered to reflect spontaneous locomotor activity [54].

#### Morris water maze training

DMS-, aCC-, and sham-lesioned mice received a total 15 days of training. Overall task performance was evaluated by calculating the time required to find the hidden platform (latency in s), average distance between the mouse and the hidden platform (mean distance to platform, cm), swimming speed (velocity, cm/s) and time spent in the 15% outer rim of the pool (thigmotaxis, %). Swimming trials were further categorized into differential search strategies according to previously described methods (see [33] for a detailed description). Briefly, three different categories of search strategy were identified ranging from proper spatial strategies (i.e., swimming directly to the platform or with one small explorative loop) to strategies that involved systematic scanning of the pool without relying on spatial information (i.e., non-spatial strategies), or those that merely consisted of repetitive looping (i.e. swimming in tight circles). Probe trials were performed after each 5 days of training (i.e. probe 1 was performed on day 6, probe 2 on day 11 and probe 3 on day 16, before continuing acquisition training) to determine whether mice actually showed a preference for the platform area as a demonstration of spatial memory. During these trials, the platform was removed from the pool, animals were allowed to swim freely during 100 s, and time spent in each quadrant was recorded.

#### Statistical analysis

All behavioural data are presented as means with standard error of the mean (SEM). Differences between mean values were determined using analysis of variance (ANOVA) procedures with Tukey test for post-hoc evaluation. Trials or trial blocks over several days between groups were analysed using repeated-measures day x group ANOVA (RM-ANOVA). Paired t-tests were used to determine learning differences between specific days within groups and one-sample t-tests when comparing the results to chance levels. Statistical analyses were performed using SPSS version 19. All statistical tests were performed with α = 0.05 (*p < 0.05, **p < 0.01 and ***p < 0.001).

### Experiment 2: DMS and aCC activation during MWM learning

#### Morris water maze training

Mice used for *in situ* hybridization experiments were trained in MWM for 2 days (2d_T, n = 7, 1 daily training session), 3 days (3d_T, n = 7, 1 daily training session) or 30 days (30d_T, n = 8, 2 daily training sessions). Free-swimming control mice (2d_SC, n = 4; 3d_SC, n = 4; and 30d_SC, n = 4) explored the same environment except that hidden platform and distal cues were removed (by placing white cardboard around the pool). Experimental (trained) and free-swimming groups were matched with respect to average time spent swimming on each trial and the total number of trials performed. Non-swimming (caged) control mice (CC, n = 5) were included that did not receive any MWM training, but were transferred between housing and training rooms together with the other mice. Heat plots depicting spatial occupancy of the pool were created by summating the swimming paths of individual mice, using custom-made MATLAB software [10,55]. These heat plots used an intensity-based pseudo-color scale to indicate spatial occupancy or dwell during a specific trial (blue low vs. red high occupancy). Notably, *Arc* expression levels in aCC and DMS were significantly correlated (Pearson r = 0.84, *p* < 0.001).

#### Quantitative in situ hybridization

*In situ Arc* hybridization was performed using previously established methods [56]. Briefly, a series of 25 μm brain sections, covering the entire rostrocaudal extent of the striatum/anterior cingulate, were collected and kept at -30°C. Tissue was postfixed in 4% (vol/vol) paraformaldehyde in 0.12 M phosphoric acid in PBS (0.1 M, pH 7.4, 30 min, 4°C; 0.9% NaCl), dehydrated (50%, 70%, 98%, 100% (vol/vol), 5 min), and delipidated (100% vol/vol chloroform, 10 min). Mouse-specific synthetic *Arc* (probe: 5’-cttgacccagcgctccaggttggcgatggtctcctggcagcggca-3’) was end-labelled with 33P-dATP (New England Nuclear) using terminal deoxynucleotidyl transferase (Invitrogen). Unincorporated nucleotides were removed using mini Quick Spin columns (Roche Diagnostics). The radioactive labelled probe was mixed with a hybridization mixture [50% (vol/vol) formamide, 4× standard saline citrate, 1× Denhardt’s solution, 10% (wt/vol) dextran sulfate, 100 μg/mL Herring sperm DNA, 250 μg/mL tRNA, 60 mM DTT, 1% (wt/vol) N-lauryl-sarcosine, and 26 mM NaHPO_4_ (pH 7.4)], applied to a series of dehydrated sections and incubated overnight at a temperature of 37°C. The next day, sections were rinsed in 1× standard saline citrate buffer at 42°C, air-dried, and apposed to an autoradiographic film (Kodak) together with a [^14^C] microscale (GE Healthcare). Films were developed 2.5 wk later in Kodak D19 developing solution and fixed in Rapid fixer (Ilford Hypam).

Autoradiographic images were scanned (CanoScan LiDE 600F; Canon), and optical densities (mean grey value per pixel) were quantified with ImageJ software (image processing and analysis in Java; National Institutes of Health). Optical density was measured in three brain sections per mouse along the rostrocaudal axis for each target region. Striatal and aCC slices were collected at +1.10 mm to +0.38 mm relative to Bregma [50]. Within striatum, we targeted the dorsomedial subregion. Mean grey values were averaged across hemispheres and brain slices, resulting in a single data point per animal for each subregion.

#### Statistical analysis

Learning-specific changes in IEG expression were evaluated by single-factor design (one-way ANOVA) in which the main effect of day of treatment (i.e., 2 days, 3 days or 30 days) on IEG expression was independently evaluated in MWM trained mice and free-swimming controls. Fisher’s LSD post-hoc tests were used for pairwise comparisons. For each subregion, learning-specific changes in IEG expression were defined as changes in IEG expression over the course of the training period. An unpaired t-test was used for the direct comparison of IEG expression present in trained and free-swimming control groups at different levels of the factor days of treatment. One-way between-groups ANOVA was used to test differences in IEG expression between the non-swimming caged control group and all experimental and control groups. Statistical analyses were performed using SigmaStat 3.1 (SYSTAT software). For all analysis, ∞ was set at 0.05 (*p < 0.05, **p < 0.01 and ***p < 0.001).

## Results

### Experiment 1

#### Brain lesion location

**Fig 1:**
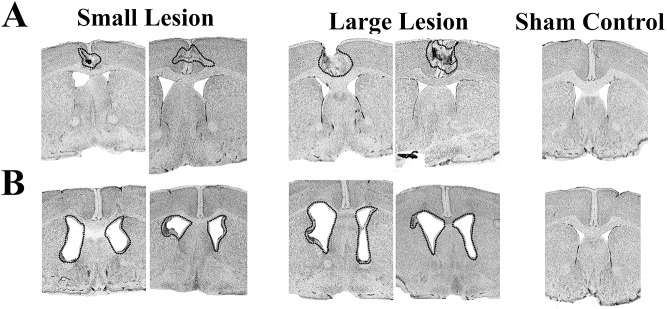
Histological verification and lesion size determination. Representative coronal sections of bilateral (A) aCC and (B) DMS lesions. The left, middle and right pictures represent small, big and sham control lesions, respectively. All lesions were restricted to the region of interest. The bilateral lesions are encircled by a dotted line.

Fig 1 shows coronal brain sections illustrating the extend of the electrolytically induced bilateral aCC (Fig 1A) and DMS (Fig 1B) lesions. ACC lesions extended between +0.98 mm and 0.00 mm, and +1.42 mm and -0.74 mm (AP to Bregma). DMS lesions extended between +1.18 mm and -0.34 mm, and +1.42 mm and -0.58 mm (AP to Bregma). Lesions were consistently between these coordinates, except for 2 animals of the DMS group, which were discarded from further analysis. Lesions in DMS were comparable in size and location with our previous work [33]. It should be noted that we aimed for comparable relative volumes of DMS and aCC damage, and that DMS lesions corresponded to the corticostriatal projection of aCC (based on Allen Mouse Brain Connectivity Atlas, available: http://connectivity.brain-map.org).

#### Additional behavioural assessment

**Fig 2:**
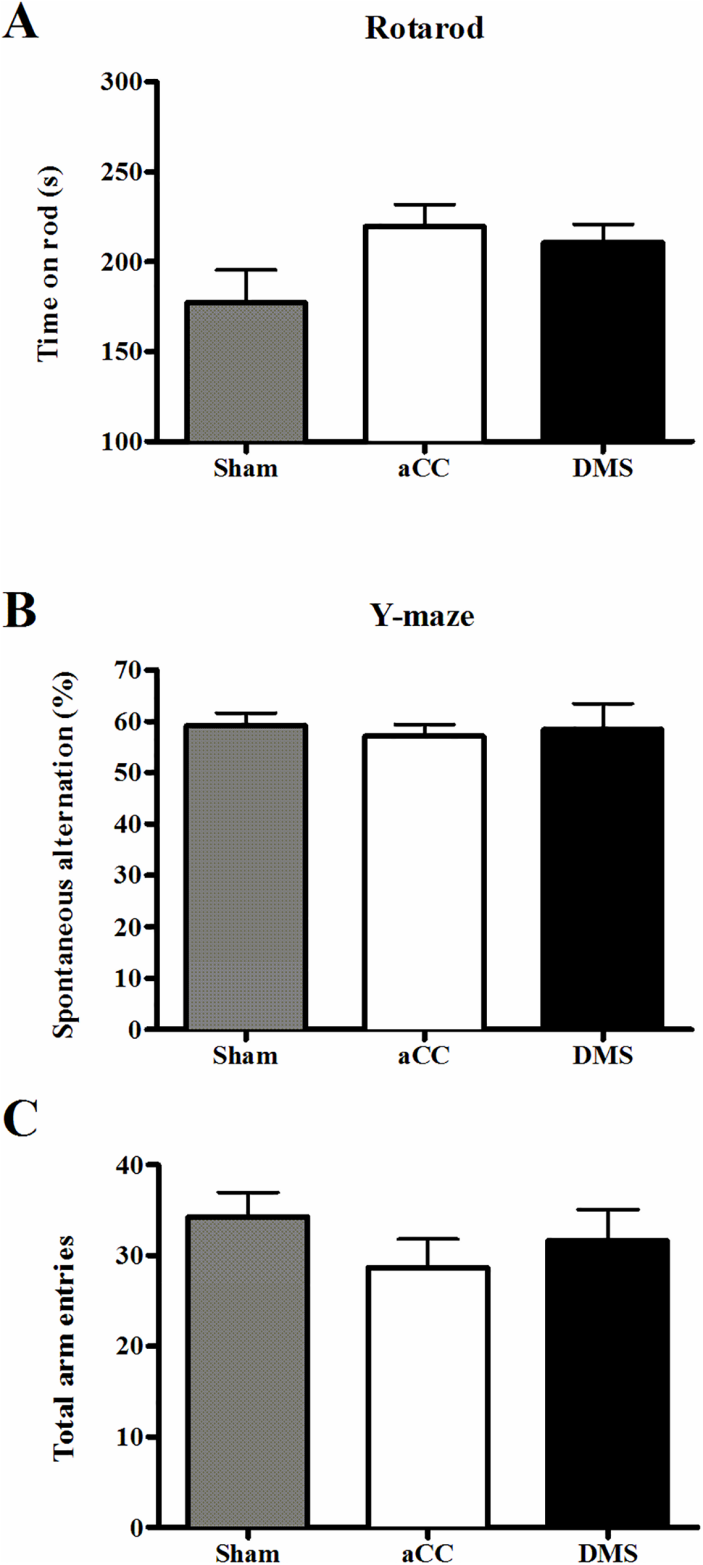
Motor performance and working memory. (A) Average fall latencies over 4 consecutive trials on the accelerated rotarod in aCC- (n = 11; white bar), DMS- (n = 9; black bar) and sham control-lesioned mice (n = 12; grey bar) indicated no motor deficits. (B) Percentage of spontaneous alternations in the y-maze showed no differences in spatial working memory between the groups, and (C) total number of arm entries showed unchanged spontaneous locomotor activity.

Motor capacity and balance were tested on an accelerating rotarod device. All animals learned to stay longer on the rod (RM-ANOVA, trial, F_3, 87_ = 7.28, *p* < 0.001) and no differences were found between lesion groups (trial x group, F_96, 87_ = 0.12, *p* = 0.99; group, F_2, 29_ = 2.50, *p* = 0.10; Fig 2A), indicating that neither aCC, nor DMS lesions caused major motor impairment. Spatial working memory was assessed by spontaneous alternation (SA) in the Y-maze. No differences in % SA were observed, which shows that spatial working memory was not impaired in the different lesion groups (ANOVA, F_2, 29_ = 0.11, *p* = 0.89; Fig 2B). Furthermore, no differences in total number of arm entries were reported between the different lesion groups (group, F_2, 29_ = 0.94, *p* = 0.40; Fig 2C), suggesting unchanged spontaneous locomotor activity.

#### MWM learning in lesioned mice

**Fig 3:**
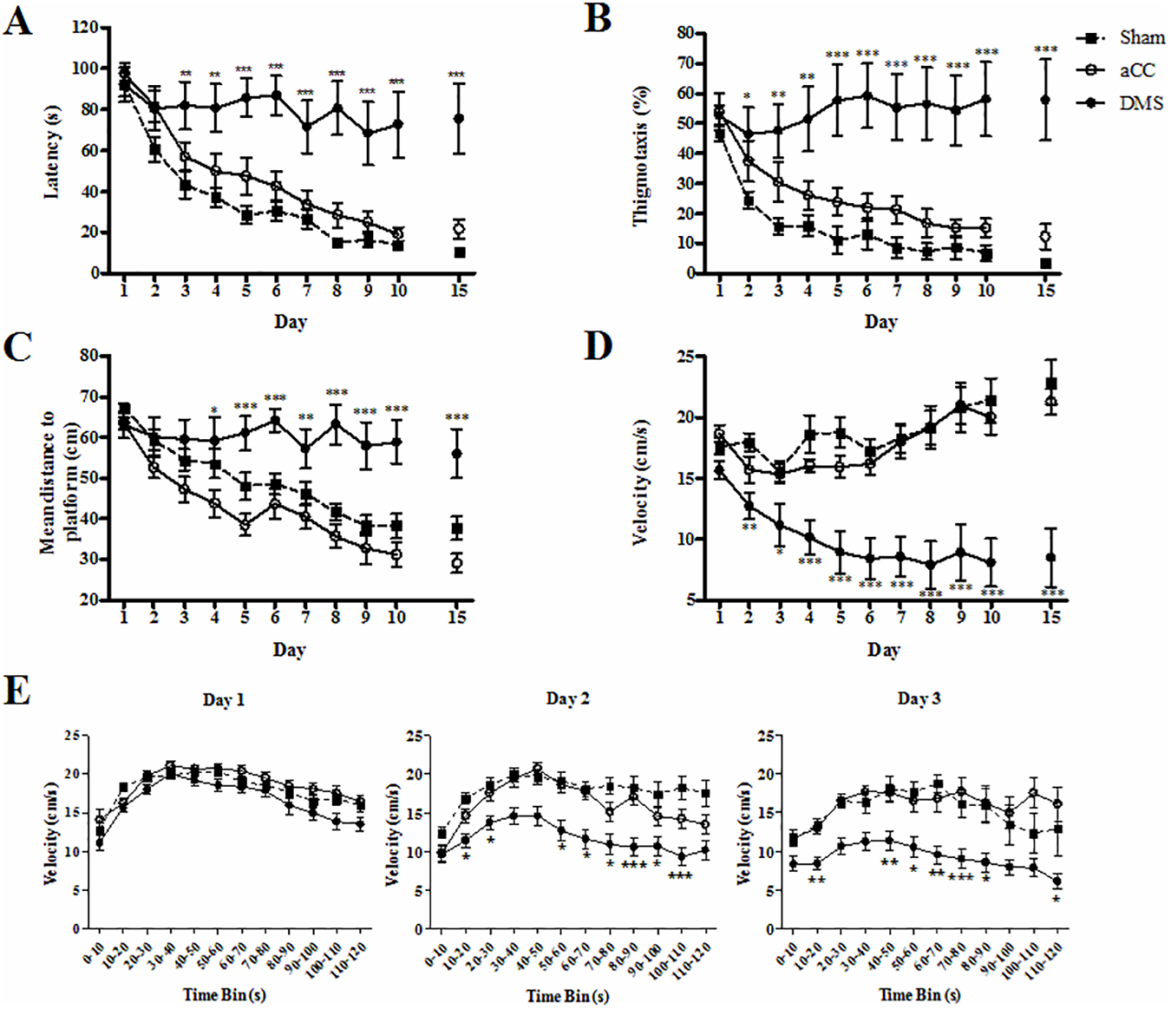
Spatial learning in the Morris water maze test. Animals with lesions in DMS (n = 9; filled circles, black bar) (A) required significantly more time to locate the hidden platform, (B) spent more time along the walls of the pool, (C) searched further away from the location of the hidden, and (D and E) swam slower from the second day of training compared to sham control group (n = 12; filled squares, grey bar). No overall differences between aCC (n = 11; empty circles, white bar) and sham control group were reported. Represented data are expressed as means ±SEM. * indicates significant differences between the sham control and lesion groups: *p < 0.05, **p < 0.01, ***p < 0.001.

The effects of lesions in aCC and DMS on spatial memory were assessed over 15 training days in the hidden platform MWM. Over the course of training, significant differences in latency decline were observed between the different groups (latency, Fig 3A, RM-ANOVA, day x group, F_28, 406_ = 4.90, *p* < 0.001; day, F_14, 406_ = 37.48, *p* < 0.001; group, F_2, 29_ = 14.86, *p* < 0.001). Post-hoc analysis revealed that DMS-lesioned mice required significantly more time to locate the hidden platform from day 3 onwards compared to the sham lesion group (all *p*-values < 0.01), and that this difference increased as training progressed (day 15; *p* < 0.001). All animals reached asymptotic performance around 10–15 days (day, F_4, 116_ = 0.16, *p* = 0.96; group, F_2, 29_ = 15.34, *p* < 0.001; day x group, F_8, 116_ = 1.40, *p* = 0.21), therefore training was limited to 15 days. Consequent to the main group and interaction effects, we compared both lesion groups against the control group. First, we found that no learning occurred in the DMS group over 15 days of acquisition training (day, F_14, 112_ = 1.11, *p* = 0.36). On the other hand, when we compared the latency curves of animals with aCC lesions and sham control animals, we did observe differences in latency curves between those groups. Both aCC-lesioned animals and sham controls were able to learn the position of the hidden platform, whereas animals with damage in aCC needed more time to do so (day, F_14, 294_ = 62.53, *p* < 0.001; group, F_1, 21_ = 5.33, *p* < 0.05; day x group, F_14, 294_ = 0.77, *p* = 0.70).

The significant interaction effect of thigmotaxis indicated that not all animals showed a typical decrease in time spent along the wall of the pool, as training progressed (Fig 3B, day x group, F_28, 406_ = 8.08, *p* < 0.001; day, F_14, 406_ = 13.89, *p* < 0.001; group, F_2, 29_ = 15.15, *p* < 0.001). Post-hoc comparison between groups indicated that animals in the DMS group spent overall more time along the wall of the pool compared to animals of the other groups (all *p*-values < 0.001), which differed significant from sham controls from day 2 onwards (*p* < 0.05). Again in pairwise comparison, we found that the aCC group also showed more thigmotaxic behaviour compared to sham animals (day, F_14, 294_ = 38.39, *p* < 0.001; group, F_1, 21_ = 4.92, *p* < 0.05; day x group, F_14, 294_ = 0.46, *p* = 0.95).

Not all animals learned to focus their searching more accurately on the platform location (distance to platform, Fig 3C, day x group, F_28, 406_ = 5.04, *p* < 0.001; day, F_14, 406_ = 29.30, *p* < 0.001; group, F_2, 29_ = 14.00, *p* < 0.001). The DMS group consistently searched farther from the location of the hidden platform compared to the other groups (post-hoc, all *p*-values < 0.01). This difference was significant compared to the sham control group from day 4 onwards (*p* < 0.05). Pairwise comparisons revealed that animals with aCC lesions on the other hand were swimming closer to the platform location compared to sham control animals (day, F_14, 294_ = 45.19, *p* < 0.001; group, F_1, 21_ = 5.56, *p* < 0.05; day x group, F_14, 294_ = 0.35, *p* = 0.99).

The ability of animals to locate the hidden platform significantly affected their swimming speed (day x group, F_28, 406_ = 6.46, *p* < 0.001; velocity, Fig 3D, day, F_14, 406_ = 5.78, *p* < 0.001; group, F_2, 29_ = 18.74, *p* < 0.001). Animals in the aCC and sham control group significantly increased their swimming speed by the end of training, whereas swimming speed in the DMS group decreased (day 1 compared to day 15, all *p*-values < 0.05). Note that the difference in velocity between DMS and sham control group was only present from day 2 (*p* < 0.05). No differences in swimming speed were present between aCC-lesioned and sham control animals when removing the DMS group from analysis (day, F_14, 294_ = 12.22, *p* < 0.001; group, F_1, 21_ = 0.33, *p* = 0.57; day x group, F_14, 294_ = 1.05, *p* = 0.40). We also calculated the mean velocity of the animals in 10 s time bins to evaluate swimming speed throughout the acquisition trials (Fig 3E). Complete data sets were analysed for the first three days only. These analyses further confirmed that all animals showed comparable swimming during the first day of acquisition training (time bin, F_11, 319_ = 32.58, *p* < 0.001; group, F_2, 29_ = 3.27, *p* = 0.05; time bin x lesion, F_22, 319_ = 1.08, *p* = 0.37; Fig 3E, left). Differences between DMS-lesioned animals and the other groups only arise from day 2 (Fig 3E, middle). All animals started trial swimming comparably (time bin, F_11, 319_ = 17.88, *p* < 0.001; group, F_2, 29_ = 7.09, *p* < 0.01; time bin x lesion, F_22, 319_ = 1.56, *p* > 0.05), although animals with DMS lesions remained slower throughout the rest of the trial compared to the sham control group (post-hoc, *p* < 0.01). This difference was even bigger from day 3 onward (time bin, F_11, 319_ = 14.45, *p* < 0.001; group, F_2, 29_ = 8.70, *p* ≤ 0.001; time bin x lesion, F_22, 319_ = 1.31, *p* = 0.16), supporting the overall decrease in swimming speed that was found after lesions in DMS.

Swimming paths of the animals during acquisition training were categorized according to 3 main search strategies (Table 1; spatial, non-spatial or repetitive). On day 5, aCC- (48%) and DMS-lesioned animals (53%) displayed a preference for repetitive looping, whereas sham control animals rather mixed the 3 main search strategies. By day 10, both the aCC (75%) and sham (75%) group developed a preference for spatial search strategies to locate the hidden platform, which further increased to 85% at the end of training for sham animals, but even dropped slightly (to 66%) in animals with aCC damage. Animals with DMS lesions on the other hand completely failed to display such a preference (D10 = 22%; D15 = 25%) and continued to rely on repetitive looping strategies (D10 = 67%; D15 = 61%). More specifically, DMS-lesioned animals primarily relied on peripheral looping strategies (Table 2; 64%) on the first day of training and progressed to circling strategies after the first week of training (D5 = 39%; D10 = 51%; D15 = 53%).

**Table 1:** Search strategies to locate the hidden platform.

		Day of training		
Main search strategy	Lesion group	5	10	15
Spatial	aCC	29	75**	66**
	DMS	11*	22	25
	Sham	33	75**	86***
Non-spatial	aCC	23	16*	20
	DMS	36	11**	14*
	Sham	33	19*	8***
Repetitive looping	aCC	48*	9***	14**
	DMS	53	67*	61
	Sham	33	6***	6***

The different search strategies over the trial blocks of day 5, 10 and 15 were categorized to one of the three main categories (spatial, non-spatial and repetitive looping) for each lesion group. Values represent the percentage of each strategy applied. * indicates a significant preference for a search strategy (compared to 33.33% chance level)

*p < .05

**p < .01

***p < .001.

**Table 2:** Development of repetitive looping strategies over the course of training.

		Day of training			
Repetitive Looping strategies	Lesion Group	1	5	10	15
Chaining	aCC	2**	26	7	14
	DMS	0#	6	8	0#
	Sham	2***	23	6	2***
Peripheral looping	aCC	64***	11	0#	0#
	DMS	42*	8	8	8
	Sham	31**	0#	0#	0#
Circling	aCC	5	11	2***	0#
	DMS	8	39*	51**	53*
	Sham	6	10	0#	4

Chaining, peripheral looping and circling strategies over the trial blocks of day 1, 2, 3, 5, 10 and 15 are represented for each lesion group. Values represent the percentage of each strategy applied. * indicates a significant preference for a search strategy (compared to 11.11% chance level)

*p < .05

**p < .01

***p < .001

# not calculated.

**Fig 4:**
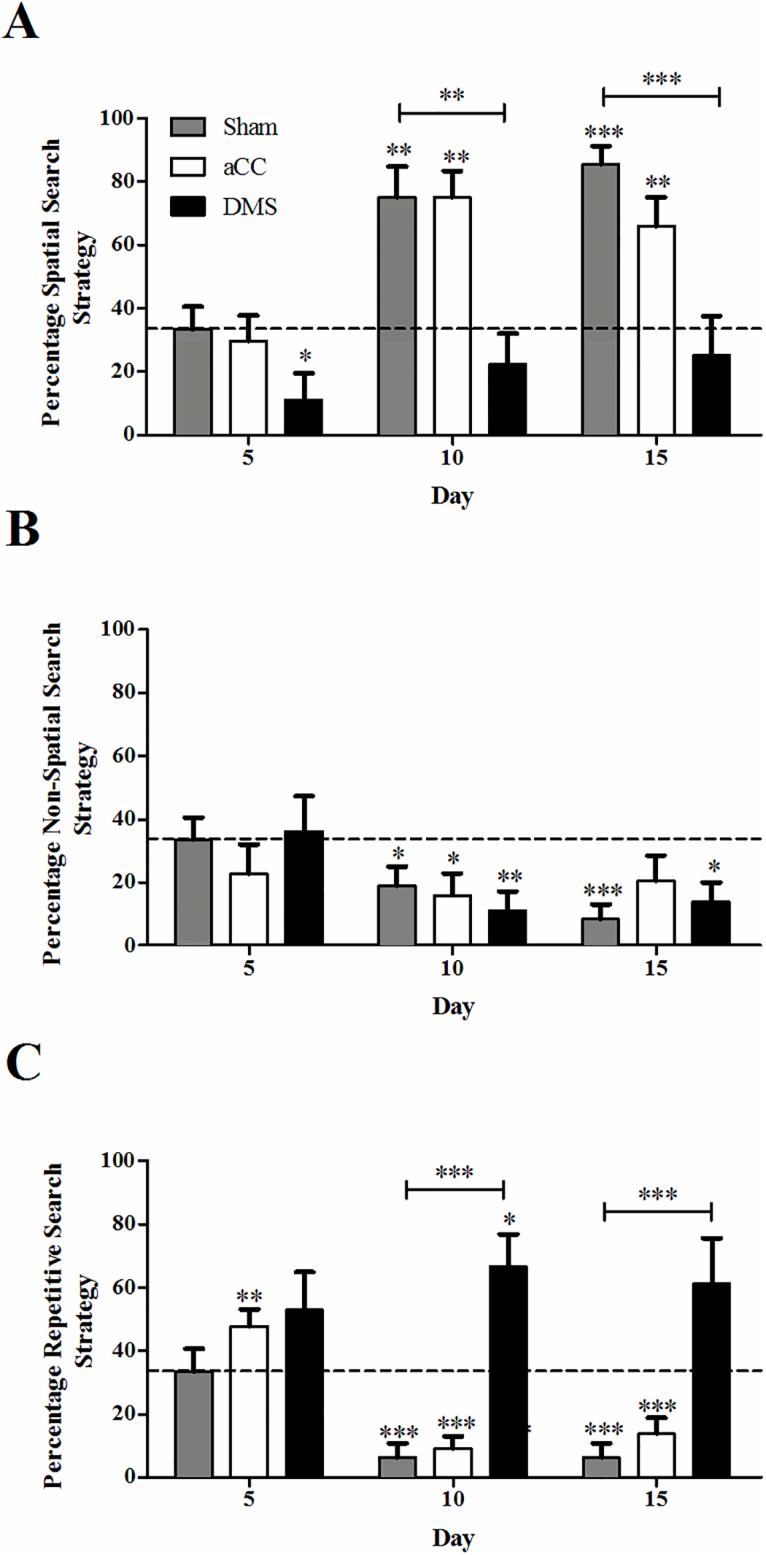
Employment of search strategies to locate the hidden platform. The strategy to search for the hidden platform location changed over the course of training and differed between lesion groups. The DMS group (black bar) failed to deploy a preference for the spatial search strategy, and instead relied on a repetitive search strategy. Represented data are expressed as means ±SEM. * indicates significant differences between the sham control and lesion groups: *p < 0.05, **p < 0.01, ***p < 0.001.

Next, we examined how spatial strategy use changed with time. When comparing the results of day 5, 10 and 15 (Fig 4), deployment of spatial search strategies over the course of training differed between lesion groups (Fig 4A; day x group, F_4, 58_ = 3.47, *p* < 0.05; group, F_2, 29_ = 5.46, *p* < 0.05; day, F_2, 58_ = 29.57, *p* < 0.001). No differences were present between aCC or sham controls at any time point (all *p*-values ≥ 0.26), whereas the DMS group used significantly less spatial strategies compared to the other groups from day 10 (all *p*-values < 0.001). In fact, spatial strategy use in the DMS group increased slightly, but non-significantly (p = 0.14) from 11% on day 5 to 25% on day 15, further confirming that this group of animals was severely impaired in their ability to learn to use spatial information to locate the hidden platform. Logically, deployment of spatial strategies coincided with decreasing use of non-spatial (group, F_2, 29_ = 0.004, *p* = 0.99; day, F_2, 58_ = 5.67, *p* < 0.01; day x group, F_4, 58_ = 1.15, *p* = 0.34; Fig 4B) and repetitive strategies (day x group, F_4, 58_ = 10.30, *p* < 0.001; group, F_2, 29_ = 14.08, *p* < 0.001; day, F_2, 58_ = 10.29, *p* < 0.001; Fig 4C). Except in the DMS group, which continued to rely predominantly on repetitive looping strategies compared to aCC and sham controls (all *p*-values < 0.05).

**Fig 5:**
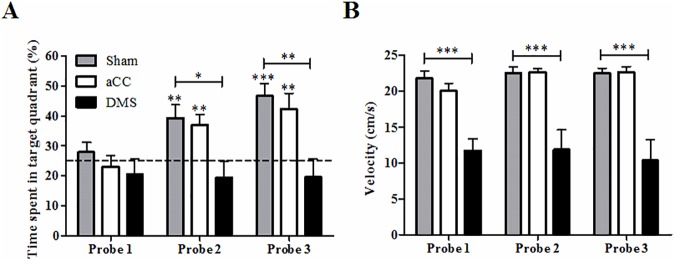
Probe trial performance in the Morris water maze test. (A) No preference for the target quadrant was present during the first probe trial (compared to 25% chance level, dotted line). During the second probe trial all groups, except animals with DMS lesions (n = 9; black bar), spent significantly more time in the target quadrant, which increased even more during the third probe trial for the sham control (n = 12; grey bars) and aCC lesion group (n = 11; white bars). (B) Animals with DMS lesions showed slower swimming speed during all probe trials. Represented data are expressed as means ±SEM. * indicates significant target preference compared to chance level and significant differences between the sham control and lesion groups: *p < 0.05, **p < 0.01, ***p < 0.001.

After each 5 days of acquisition training, spatial accuracy was studied by removing the platform from the pool during probe trials (Fig 5A). The first probe trial did not show differences between groups for time spent in the target quadrant (ANOVA, F_2, 29_ = 1.11, *p* = 0.34; Fig 5A), and student’s t-test (Bonferroni correction) indicated that none of the groups spent significantly more than 25% of the time (i.e., chance level) in this quadrant (all *p*-values > 0.34). Differences between the different groups were present from the second probe trial (probe 2, F_2, 29_ = 5.88, *p* < 0.01; probe 3, F_2, 29_ = 8.07, *p* < 0.01). Post-hoc analysis indicated that the DMS group spent less time in the target quadrant compared to the aCC and sham control group in both probe trials (all *p*-values < 0.01). Animals with lesions in the DMS also failed to spend more than 25% search time in this quadrant (probe 2, t_8_ = -1.14, *p* = 0.29), even after 15 days of training (probe 3, t_8_ = -.93, *p* = 0.38). Furthermore, swimming speed during probe trials differed between lesion groups from the first probe trial onwards (probe 1, F_2, 29_ = 17.62, *p* < 0.001; probe 2, F_2, 29_ = 16.26, *p* < 0.001; probe 3, F_2, 29_ = 18,72, *p* < 0.001; Fig 5B), verifying that animals with lesions in DMS overall showed slower swimming speed compared to aCC and sham controls (all *p*-values < 0.001).

### Experiment 2

#### MWM learning in WT mice

**Fig 6:**
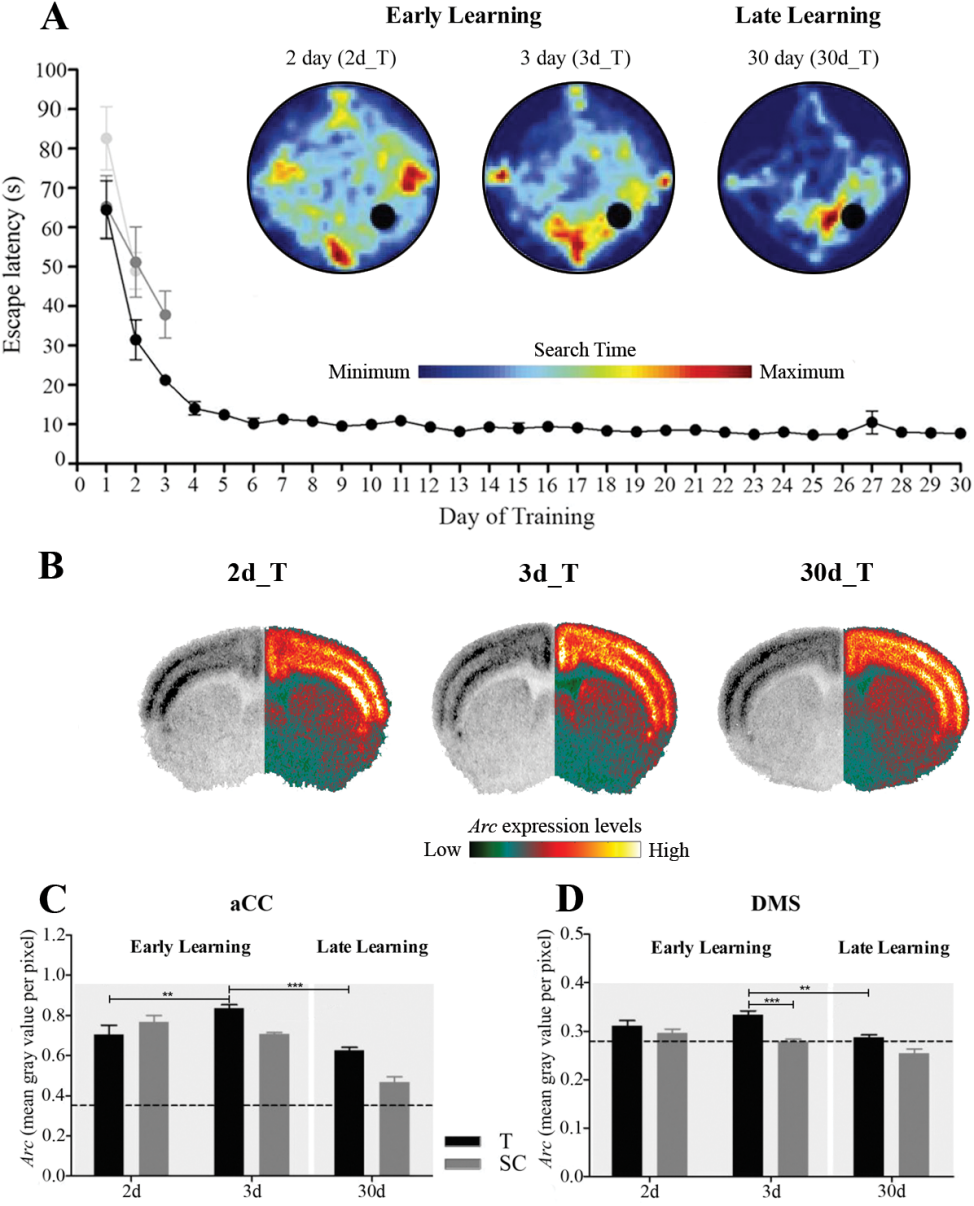
Learning-dependent changes in *Arc* expression. (A) Learning curves, demonstrating task acquisition as a decrease in latency (s) before reaching the hidden platform, of 2 days (2d_T), 3 days (3d_T) and 30 days (30d_T) trained mice are light grey, medium grey and black, respectively. Heat plots (blue and red indicating minimum and maximum search time spent, respectively) during early learning in the 2d_T and 3d_T group indicate that the overall search area remains variable and covers most of the environment with some focus towards the platform quadrant after 3 days of learning. During the late learning phase search patterns in the 30d_T group are highly focused on the hidden platform. Black circle represents the hidden platform. (B) Coronal sections displaying *Arc* expression in trained mice during early learning (2d_T and 3d_T) and late learning (30d_T) group. The left hemisphere shows the original autoradiogram in grey scale and the right hemisphere shows its matched pseudo-colour counterpart (dark green to white indicating no signal to maximum signal, respectively). (C) *Arc* expression in aCC increased from 2-day to 3-day trained group and decreased upon overtraining. In free-swimming controls, a reduction in *Arc* expression was present from the early to the late phase. At 3 days, trained mice demonstrated significantly higher *Arc* expression compared to free-swimming controls. (D) During the early learning phase *Arc* expression in DMS of trained mice was upregulated, albeit borderline significant, while extensive training resulted in a decreased *Arc* expression level. Free-swimming controls only showed a reduction in *Arc* expression from the early to the late phase. At 3 days, *Arc* expression in trained mice was significantly higher than in free-swimming controls. Dotted line represents baseline *Arc* expression level of cage control animals. Black and grey bars represent experimental (trained, T) and free-swimming control (SC) groups, respectively. Represented data are expressed as means ±SEM. * indicates significant differences between groups: **p < 0.01, ***p < 0.001.

All animals were trained for 2, 3 or 30 days. Obviously, MWM performance in the 2-day group (2d_T) was characterized by an unfocused search pattern that covered the entire search area (Fig 6A), whereas the search pattern of the 3-day group (3d_T) was more goal-directed, but still variable. In contrast, the search pattern of the 30-day (30d_T) extensively trained group was highly focused on the hidden platform location. Similar to experiment 1, latency (Fig 6A) decreased during the early learning phase between day 1 and 2 in the 2-day group (t_1,6_ = 3.97, *p* < 0.01) and between day 1 and 3 in the 3-day group (F_2,12_ = 4.06, *p* < 0.05). Furthermore, time to locate the hidden platform decreased significantly over the course of training (day, F_29,203_ = 33.82, *p* < 0.001), reaching asymptotic performance around 10–15 days. Comparing between the training groups showed that the 30-day group obviously performed much better than the 2-day and 3-day groups (F_2,19_ = 27.24, *p* < 0.001), whereas no significant change occurred between 2 and 3 days. These results confirm that 2-day and 3-day trained groups represent the flexible, early learning phase, whereas the 30-day group reflects more stable performance typical of the habitual phase.

#### Arc immediate early gene expression

*Arc* expression in aCC (T, F_2,18_ = 12.46, *p* < 0.001; SC, F_2,9_ = 40.20, *p* < 0.001; Fig 6B) and DMS (T, F_2,18_ = 7.55, *p* < 0.01; SC, F_2,9_ = 7.23, *p* < 0.05; Fig 6C) was upregulated above the non-trained baseline level. *Arc* expression in aCC (Fig 6B and 6C) showed a significant increase in 2- and 3-days trained mice (*p* < 0.01) that was absent in free-swimming controls (post-hoc test, 2d_SC vs 3d_SC, *p* = 0.12), resulting in significantly higher *Arc* expression in trained compared to free-swimming mice at 3 days (t_1,9_ = 5.16, *p* < 0.001). *Arc* expression declined in the late phase in both trained mice (post-hoc test, 3d_T vs 30d_T, *p* < 0.001) and free-swimming controls (post-hoc tests, 2d_SC vs 30d_SC and 3d_SC vs 30d_SC, *p* < 0.001).

Similarly, *Arc* expression in DMS (Fig 6B and 6D) was upregulated in trained groups during the early learning phase (post-hoc test, 2d_T vs 3d_T, *p* = 0.07), but not in the free-swimming controls (post-hoc test, 2d_SC vs 3d_SC, *p* = 0.13). As a result, *Arc* expression at 3 days was significantly higher in trained compared to free-swimming animals (t_1,9_ = 4.91, *p* < 0.001). Following extended training, both trained (post-hoc tests, 2d_T vs 30d_T, *p* < 0.01; 3d_T vs 30d_T, *p* = 0.06) and free-swimming mice (post-hoc test, 2d_SC vs 30d_SC, *p* < 0.01) displayed a decrease in *Arc* expression.

## Discussion

We examined the involvement of aCC and DMS in spatial cognition, employing the MWM task that has been the dominant paradigm in rodents for over three decades [2–4,9]. Although this test is mostly viewed as a hippocampus-dependent task [1,57], other telencephalic regions such as mPFC and dorsal striatum (part of a functional corticostriatal system) also appear to play a crucial, but less understood role in this type of learning [10]. To compare aCC and DMS involvement directly, we performed electrolytic lesions that selectively targeted these brain regions, and demonstrated that DMS lesions severely impaired MWM performance. DMS-lesioned animals displayed impaired learning, increased thigmotaxis, and decreased search precision. Also, these animals failed to show preference for the target quadrant relative to the other quadrants during the probe trials.

Even though dorsal striatum has been classically related to motor control [58], it seems unlikely that the effects of DMS lesions observed here were essentially caused by sensorimotor impairment. Firstly, several studies reported that sensorimotor functions were affected by lesions in dorsolateral striatum (DLS), rather than DMS [5,6,10,59,60]. Furthermore, our results demonstrate that DMS lesions did not affect spontaneous locomotor activity as measured in the Y-maze, nor affected motor coordination and equilibrium in the rotarod task. They even showed a tendency to stay longer on the rotating rod compared to sham controls. Moreover, there was no difference in swimming speed in DMS-lesioned mice during the first day of MWM training. Reduced swimming speed at a later phase might have resulted from their inability to find the platform, and ensuing extinction of active escape strategies.

In accordance with our previous findings [33], animals with DMS lesions showed increased thigmotaxis and more dispersed searching for the hidden platform. Devan and colleagues [5] also reported thigmotaxis in rats, but only during early trials, whereas Lee and colleagues [32] failed to find thigmotaxic swimming in mice. We, on the other hand, observed thigmotaxis during the entire course of training, possibly due to the fact that, in our study, the medial part of the striatum was specifically targeted. Thigmotaxis has been implicated as a symptom of altered search strategy and impaired goal-directed learning [5,33]. Indeed, our search strategy analyses confirm that DMS-lesioned mice were unable to employ goal-directed learning strategies, and relied predominantly on repetitive looping instead of spatial searching. Consequently, these animals failed to display preference for the platform position during the probe trials, even after 15 days of training.

On the other hand, aCC damage affected spatial cognition more subtly, but notwithstanding conflicting literature reports, we observed significantly impaired spatial cognition in aCC-lesioned mice using detailed behavioural analysis of MWM performance. We observed slightly slower learning curves, increased thigmotaxis, and decreased search precision in this group compared to controls. Search strategy analyses were consistently inflexible and erratic deployment of goal-directed spatial search strategies in these mice. Whilst sham controls flexibly switched between the different strategies, aCC animals preferred more repetitive strategies during early learning. Furthermore, although the aCC group showed a preference for spatial search strategies from the second week, they were less able than control animals to maintain stable use of these strategies. These detailed analyses qualify previous lesion studies that failed to report aCC involvement in spatial task acquisition [24–26]. We therefore conclude that both DMS and aCC lesions impair the cognitively demanding deployment of spatial search strategies, although to a different extent.

In addition, elevated *Arc* expression in both aCC and DMS during early MWM learning indicate that both brain regions are involved in goal-directed, early spatial learning. Anatomical connectivity between these regions has been established (Allen Mouse Brain Connectivity Atlas, available: http://connectivity.brain-map.org), whereas our present observations suggest that these brain regions also play a joint role in spatial cognition. Consistent with our observation that aCC lesions affect MWM learning in a different way from DMS lesions, studies using different methodology indicated that this involvement might in fact be functionally dissociated. Indeed, aCC has been suggested to have a more general, executive function and several studies implicated this brain structure in effort-based decision making [61–64]. For example, it has been suggested that aCC controls cost-benefit decisions about the possible course of action. Consequently, Walton and Mars [65] observed a higher firing rate in aCC during actions that maximized overall reward gain by encoding a cumulative history of recent rewards. Such a function of aCC could definitely be convergent with our observations that aCC is important for deployment of efficient navigation strategies. During the early stages of spatial learning, mPFC areas such as aCC might be involved in cost-benefit choices between competing strategies. Spatial strategies are definitely the most cognitively effortful ones (high cost), but could deliver the most economical yield in terms of successful platform situation (high benefit), compared to strategies with lower success rates, which is also in accordance with the putative error prevention role of the aCC [66,67] and the role of mPFC in task difficulty [68].

The current study aimed to investigate the differential role of the anatomically connected aCC and DMS in early phases of learning and memory. Brain lesion experiments demonstrated impaired learning curves and inability to deploy spatial search strategies in mice with DMS damage in the absence of other major motor or working memory impairments, whereas aCC lesions had a more subtle effect. In addition, expression of the neuroplasticity-related, IEG *Arc* suggested the involvement of aCC and DMS in goal-directed, early phases of MWM learning. It must be noted that more studies are needed to further investigate the significance of aCC activation in relation to DMS. To determine if early spatial learning and memory depends on the integrity of circuits connecting aCC and DMS, one might investigate whether increased *Arc* expression in aCC is also observed in trained DMS-lesioned animals, or use disconnection lesions or reversible activation studies to disrupt communication between brain regions [69,70].

## Supporting information

S1 Dataset Dataset. (XLS)
